# Application of Reinforcement Learning Methods Combining Graph Neural Networks and Self-Attention Mechanisms in Supply Chain Route Optimization

**DOI:** 10.3390/s25030955

**Published:** 2025-02-05

**Authors:** Yang Wang, Xiaoxiang Liang

**Affiliations:** 1 Department of Statistics, Pennsylvania State University, University Park, PA 16802, USA; 2 Business Analytics, Washington University in St. Louis, St. Louis, MO 63130, USA; l.xiaoxiang@wustl.edu

**Keywords:** supply chain optimization, graph neural networks, self-attention mechanism, meta-reinforcement learning, dynamic route planning

## Abstract

Optimizing transportation routes to improve delivery efficiency and resource utilization in dynamic supply chain scenarios is a challenging task. Traditional route optimization methods often struggle with complex supply chain network structures and dynamic changes, which require a more efficient and flexible solution. This study proposes a method that integrates Graph Neural Networks (GNNs), self-attention mechanisms, and meta-reinforcement learning (Meta-RL) in order to address route optimization in supply chains. The goal is to develop a path planning method that excels in both static and dynamic environments. First, GNNs model the supply chain network, converting node and edge features into high-dimensional graph representations in order to capture local and global network information. Next, a Transformer-based strategy network captures global dependencies, optimizing path planning. Finally, Meta-RL enables rapid strategy adaptation to dynamic changes (e.g., new demand points or route disruptions) with minimal sample support. Experiments on multiple supply chain datasets show that our method improves path planning quality by about 7%, compared to traditional methods, achieving a path coverage of 92.29%. Ablation studies reveal that the on-time delivery rate improves by nearly 30% over the baseline model. These results demonstrate that the proposed method not only optimizes routes but also significantly enhances the overall efficiency and robustness of supply chain networks. This research provides an efficient route optimization framework applicable to complex supply chain management and other scheduling fields, offering new insights and technical solutions for future research and applications.

## 1. Introduction

With the acceleration of globalization and the continuous rise in logistics demand, the complexity and dynamism of supply chains have significantly increased [1,2]. As one of the core tasks of supply chain management, transportation route optimization plays a crucial role in reducing operational costs, improving delivery efficiency, and enhancing customer satisfaction [3]. However, traditional route planning methods, such as rule-based optimization algorithms [4,5] and static models, often struggle to adapt quickly and maintain high optimization performance in dynamic scenarios such as the addition of new demand points or route disruptions. Therefore, developing adaptive and robust route optimization methods has become a critical research direction in the field of supply chain management.

Currently, supply chain route optimization primarily relies on classical operations research methods [6,7] and heuristic algorithms [8]. Operations research methods, including Linear Programming (LP) and Integer Programming (IP) [9], aim for precise solutions and are well-suited for static and small-scale supply chain problems [10]. These methods clearly describe variables and constraints through mathematical formulations, with objective functions typically focused on minimizing transportation costs or maximizing resource utilization efficiency. However, as the problem scale expands and dynamic requirements emerge, the computational complexity of traditional optimization methods increases rapidly, leading to reduced efficiency in practical applications. Moreover, their limited flexibility in adapting to dynamic changes restricts their applicability in real-world complex supply chains.

To address the low computational efficiency of traditional methods, researchers have proposed various heuristic algorithms such as Genetic Algorithm (GA) [11], Particle Swarm Optimization (PSO) [12], and Simulated Annealing (SA) [13]. These algorithms simulate natural optimization processes to find near-optimal solutions within a limited time, which offer strong adaptability and flexibility. However, they rely heavily on well-designed heuristic rules, and the quality of the solutions is largely constrained by initial parameters and algorithm configurations. In large-scale and dynamically changing supply chain scenarios, heuristic algorithms still face limitations, particularly in balancing the trade-off between the accuracy of the global optimum and the convergence speed.

With the rise of machine learning, Graph Neural Networks [14] have gained attention for their powerful ability to model graph structures. In supply chain optimization, GNNs are employed to model the relationships between nodes and edges in supply chain networks, capturing both local neighborhood information and global dependencies in order to optimize route planning tasks. For example, GNNs can embed features of nodes (e.g., warehouses, customer points) and edges (e.g., transportation time, cost) within supply chains to generate high-dimensional representations that guide route planning decisions [15]. Additionally, GNN variants such as Graph Attention Networks (GATs) introduce attention mechanisms [16], further improving performance in complex network scenarios. While the application of GNNs in supply chain optimization is still in its early stages, their capabilities have already demonstrated significant potential.

In recent years, self-attention mechanisms have been increasingly introduced into the field of reinforcement learning due to their outstanding performance in capturing long-range dependencies in sequential data [17,18]. In supply chain optimization problems, reinforcement learning models incorporating self-attention mechanisms can effectively model global information within complex network structures, optimizing route planning strategies [19,20]. For instance, the ARiADNE system utilizes attention-based neural networks to model the supply chain environment [21], significantly enhancing strategy learning efficiency. Compared to traditional reinforcement learning, self-attention mechanisms are better suited to handling complex dependencies in multi-objective optimization problems. However, these methods still exhibit shortcomings in dynamic adaptation and rapid generalization capabilities, which offer room for further integration with other advanced technologies.

Although existing methods have achieved some success in specific scenarios, the dynamic, diverse, and large-scale nature of complex supply chain networks continues to impose higher demands on optimization techniques. Traditional optimization methods and heuristic algorithms exhibit significant limitations when dealing with dynamic changes and large-scale problems. While machine learning methods have improved modeling and optimization efficiency, their constraints in global information modeling and dynamic adaptation capabilities remain unresolved. Meanwhile, further optimization of supply chain management not only reduces enterprise costs but also has profound implications for resource utilization and environmental sustainability, thus providing strong motivation for the proposed research.

This research aims to combine the advantages of Graph Neural Networks, self-attention mechanism (Transformer), and reinforcement learning in order to propose an efficient and highly adaptive supply chain route optimization method. The goal is to achieve global optimization of route planning and enhance dynamic adaptability in complex and dynamic supply chain networks by constructing a reinforcement learning framework that integrates GNNs and Transformers. Compared to the existing methods, our framework has the following significant advantages: First, the GNN-based supply chain network representation can effectively capture complex network relationships, significantly improving the global optimization capability of the strategy. Second, the introduction of the Transformer further strengthens the modeling of long-range dependency information, improving the efficiency and accuracy of strategy learning. Third, through meta-reinforcement learning, our method demonstrates strong rapid adaptation in dynamic scenarios, which enables quick adjustments in strategy when facing unexpected events such as new demand points or route disruptions.

In summary, this research provides an innovative solution to the important issue of supply chain route optimization by integrating GNNs, Transformers, and reinforcement learning. The framework not only significantly improves the efficiency and accuracy of route planning but also possesses rapid adaptation capabilities in dynamic scenarios, thereby providing important technical support for resource utilization optimization and cost control in supply chain management. More importantly, our method has good scalability and can be applied to other complex scenarios that require network optimization, such as traffic network scheduling and logistics delivery planning. The results of this research provide a new perspective on solving supply chain optimization problems and are expected to play an important role in improving enterprise operational efficiency, resource allocation, and sustainable development.

The contributions of this paper can be summarized in the following three aspects:

1. Graph Neural Networks: This paper applies GNNs to supply chain route optimization, using it to capture local and global network features. By embedding node (e.g., location, demand) and edge (e.g., transportation time, cost) features into high-dimensional graph representations, GNNs effectively model complex relationships and adapt to dynamic scenarios, providing valuable inputs for strategy learning.

2. Transformer Model: A strategy network based on the Transformer is built, leveraging GNN-generated graph representations to capture global dependencies. The self-attention mechanism optimizes node interactions and handles multi-objective optimization with time window constraints, enhancing path planning quality and on-time delivery rate.

3. Meta-Reinforcement Learning (Meta-RL): A fast adaptation strategy based on Meta-RL is designed to address demand fluctuations and path disruptions. Through meta-training on historical tasks, the model quickly optimizes strategies in dynamic environments, reducing adaptation time, improving robustness, and offering strong support for supply chain optimization.

The structure of this paper is as follows: Section 2 introduces the research background and significance, pointing out the limitations of traditional methods in complex and dynamic supply chains, and reviews the applications of Graph Neural Networks, reinforcement learning, and meta-reinforcement learning in this field, highlighting the shortcomings of existing studies. It also clarifies the innovations of this research and its theoretical and practical value. Section 3 provides a detailed introduction to the three main methods, Graph Neural Networks (GNNs), Transformer models, and meta-reinforcement learning, which explains their basic principles, structures, and applications in supply chain optimization and demonstrates how these methods work together to achieve efficient optimization. Section 4 describes the experimental design and implementation process, including hardware and software environments, dataset characteristics, evaluation metrics, and a comparative analysis of experimental results, validating the effectiveness and performance advantages of the proposed method. Section 5 summarizes the research findings, discusses the significance and limitations of the study, and proposes future research directions such as dataset expansion, lightweight model design, introduction of interdisciplinary theories, and strengthening model interpretability research.

## 2. Related Work

In today’s complex and dynamic supply chain networks, optimizing logistics paths and managing operational efficiency have become key tasks for enterprises to maintain a competitive advantage. However, supply chain planning and management involve multi-level decision-making issues, including strategic layout, tactical planning, and operational execution [22]. With the rapid development of big data, artificial intelligence (AI), and optimization algorithms, significant progress has been made in the field of supply chain optimization, both theoretically and practically [23]. From traditional heuristic algorithms to reinforcement learning-based intelligent decision-making methods, innovations in various technologies have provided new solutions for addressing complex problems in supply chains.

In particular, in recent years, with the improvement in computational power and algorithmic advancements, researchers have increasingly focused on how to use advanced technologies to more efficiently handle uncertainty and risk. For example, the emergence of large language models [24] (LLMs) has broken down the communication barriers between humans and machines in traditional supply chain optimization; methods based on Graph Neural Networks have provided new perspectives for handling complex supply chain networks. The emergence of these technologies has not only driven the technological development of supply chain optimization but has also provided abundant exploration space for research in related fields. This chapter aims to systematically review and analyze the research progress in supply chain path optimization and related technologies, with the goal of providing reference and inspiration for future research. Next, we will review recent related studies, focusing on the latest advancements in supply chain optimization in path planning, risk management, and algorithm applications.

Shaked et al. proposed a method to introduce the attention mechanism into Graph Neural Networks (GNNs) in their paper [25]. By assigning weights to different neighbors, they effectively identified important nodes in complex networks. The advantage of this approach lies in its flexibility and precision, providing a novel approach for dynamic path planning and key node identification in supply chain networks. By leveraging the attention mechanism, it enhances the ability to identify potential bottlenecks in supply chain networks, thereby improving optimization outcomes. Li, Beibin, and others demonstrated the potential application of large-scale language models in supply chain optimization in their paper [26]. This research designed the OptiGuide framework, which uses natural language input to explain and analyze complex optimization scenarios, significantly improving the level of automation and human-machine collaboration in supply chains. This study provides insight into how to use language models to achieve semantic interpretation and transparency for complex problems while also offering the possibility for intelligent interactions in supply chain path optimization. Jalal, Aura Maria, and other researchers have systematically reviewed integrated methods for strategic, tactical, and operational decision-making in logistics network planning, revealing the interdependencies and optimization potential between decisions at different levels [27]. These studies highlight the advantages of integrated decision-making methods in improving supply chain responsiveness and reducing operational costs. However, traditional integration methods still face challenges related to data aggregation and model complexity, providing ideas for further research into dynamic optimization models. Suryawanshi, Pravin, and others systematically analyzed supply chain optimization models under risk and uncertainty conditions in a paper [28], identifying seven major research themes through network analysis. This study provides detailed insights into the robustness and resilience management of supply chains, but current models mostly focus on single scenarios or specific assumptions, inspiring research into multi-scenario and multi-variable integrated optimization. As for the paper published in recent years by Yan, Yimo, and others [29], it comprehensively reviewed the application of reinforcement learning techniques in logistics and supply chain management, pointing out that the adaptability of reinforcement learning in dynamic environments offers new tools for solving real-time decision-making problems. Especially its successful application in urban logistics and last-mile delivery provides insights for path optimization. Despite the excellent performance of reinforcement learning methods, their data requirements and the complexity of model training remain major bottlenecks. Toorajipour, Reza, and others summarized the main applications and research directions of artificial intelligence in supply chain management, particularly the contributions of AI in logistics, production, and market analysis [30]. Through a systematic literature review, this study identified technical gaps in existing research and pointed out that artificial intelligence could further enhance decision-making intelligence and efficiency in supply chains in the future.

Despite the significant progress made in methods and applications in the aforementioned studies, certain limitations remain. For instance, most studies focus on the application of single technologies in specific scenarios, lacking in-depth exploration of the integration and collaboration of multiple technologies. Additionally, many optimization models still need improvement in handling large-scale, dynamic, and uncertain data [31]. Our research, based on these shortcomings, integrates multiple technologies and optimization methods, striving to develop a more adaptable and generalizable supply chain path optimization model. This not only fills the technological gaps in current research but also brings greater value and potential for practical applications in the supply chain domain.

The relationship between our study and existing work lies in the fact that we have fully absorbed the core ideas from the aforementioned studies on path optimization, dynamic decision-making, and uncertainty management. For example, we have drawn on the information-capturing capability of Graph Neural Networks in complex network structures while also combining the dynamic adaptability of reinforcement learning to address real-time changes in the supply chain. Furthermore, the integrated approach in traditional optimization methods provides theoretical support for our multi-level optimization framework, while the semantic analysis capabilities of large-scale language models offer a novel implementation path for data preprocessing and interactive optimization. Nevertheless, compared to existing research, there are significant differences in our choice of methods and problem definition.

Existing research typically focuses on the in-depth application of a specific domain or technology but falls short in the areas of multi-technology integration and cross-scenario optimization. To address this gap, this study proposes a new framework based on ensemble learning and intelligent optimization algorithms, which utilizes Graph Neural Networks for dynamic feature extraction of supply chain network structures while combining reinforcement learning for real-time updates of optimal paths in dynamic environments. Additionally, we introduce a hybrid optimization strategy that integrates traditional mathematical programming models with machine learning algorithms, improving the model’s adaptability to large-scale, complex supply chain data. This interdisciplinary, multi-technology integration approach not only broadens the depth of optimization research but also significantly enhances the feasibility and robustness of the model in practical applications.

Compared to traditional research, the innovations of this study primarily lie in the following aspects. First, we propose a comprehensive framework for dynamic optimization, which can effectively address the diverse uncertainties in supply chains. Second, we combine Graph Neural Networks with reinforcement learning, achieving breakthroughs in the robustness and real-time capabilities of path optimization. Lastly, by applying natural language processing techniques to the field of supply chain optimization, we open new research directions in semantic analysis and interactive decision-making. These innovations make this study a strong driver both theoretically and practically.

In summary, this study builds upon the achievements of previous research, offering new solutions to existing problems and making progress in both methodological innovation and application expansion. By constructing a dynamic, intelligent optimization framework, we not only fill the research gap in multi-technology integrated optimization but also provide significant reference value for future research and applications in the supply chain field.

## 3. Methodology

**Figure 1:**
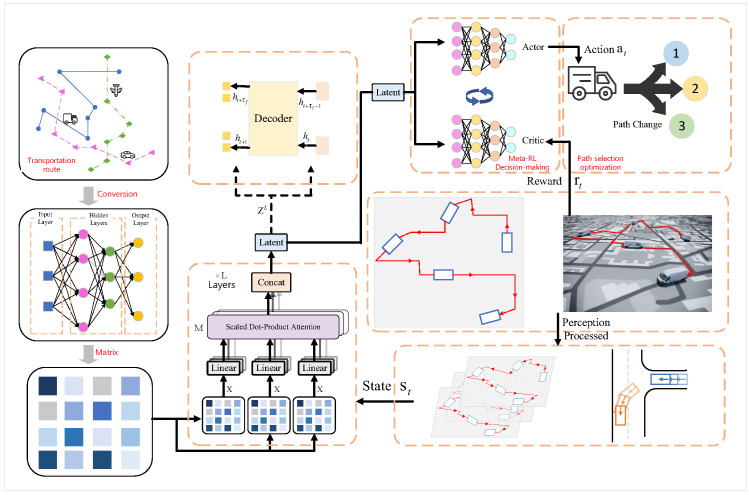
Overall algorithm flow chart.

In this chapter, we will provide a detailed introduction to the three main methods used in this study: Graph Neural Networks [32], Transformer model [33], and meta-reinforcement learning [34]. Each of these methods has unique advantages and plays a critical role in this research. Graph Neural Networks (GNNs) are effective in handling complex network structures and extracting relational information between nodes, making them suitable for dynamic path optimization in supply chain networks. The Transformer model excels in processing large-scale data and long-range dependencies through its self-attention mechanism. Meanwhile, meta-reinforcement learning provides powerful, intelligent decision support for the path optimization process by rapidly adapting to different tasks. Next, we will gradually introduce the specific applications of these methods and their roles in the algorithm of this paper and demonstrate how they work together to achieve efficient optimization through the overall algorithm flowchart. The overall algorithm flowchart is shown in Figure 1.

### 3.1. Graph Neural Networks

**Figure 2:**
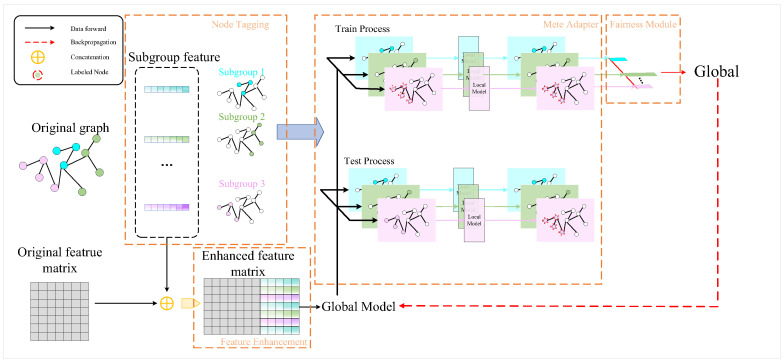
Graph Neural Network framework.

Graph Neural Networks are deep learning models used for processing graph-structured data, which are widely applied in fields such as social networks [35], knowledge graphs [36], recommendation systems [37], and supply chain path optimization. GNNs effectively capture the relationships between nodes and edges in a graph, allowing them to handle dependencies between nodes and structural information of the graph, thereby extracting meaningful features from graph data. Unlike traditional deep learning methods, GNNs can directly process non-Euclidean data structures like graphs, perform feature learning on graph nodes, and propagate information through the graph’s topology. The framework of a Graph Neural Network is shown in Figure 2.

In the integration of Graph Neural Networks and Transformer, the transmission of graph representations is a critical step. GNN first performs feature embedding on the nodes and edges of the supply chain network using graph convolution or graph attention layers, generating high-dimensional vector representations for each node. These node representations typically have a uniform dimension, $d_{\mathrm{model}}$, which precisely matches the input dimension of the Transformer, providing a foundation for subsequent processing.

Before passing the node representations to the Transformer, fine feature transformation and encoding are required. Research commonly uses sinusoidal/cosine positional encoding or learnable positional embeddings to add sequential and relative positional information to the static graph structure. This step ensures that even in complex supply chain networks, the topological relationships and relative importance of nodes are preserved. After positional encoding fusion, the node representation matrix serves as the input to the self-attention layers, enabling the Transformer to capture deeper interactions between nodes.

Once inside the Transformer, the multi-head attention mechanism comprehensively analyzes the graph node representations, using parallel attention heads to capture complex dependencies in different subspaces. To retain the local feature information of the original graph network, techniques such as residual connections and layer normalization are commonly employed. This design not only mitigates the vanishing gradient problem in deep network training but also allows the model to preserve local network features extracted by the GNN while modeling global dependencies, achieving a complementary integration of the advantages of both the GNN and Transformer.

In GNNs, the representation of each node is updated through the feature information of its neighboring nodes. Specifically, the core idea of the GNN model is to propagate information between nodes using a message passing mechanism and iteratively update the node representations. Suppose the graph G=(V,E) consists of a set of nodes *V* and edges *E*, where each node v∈V has a feature vector $h_{v}$. The core formula of the GNN is the node feature update rule:

(1) $$h_{v}^{\left(k+1\right)}=\mathrm{Update}\left(h_{v}^{\left(k\right)},\mathrm{Aggregate}\left(\left\{h_{u}^{\left(k\right)}:u\in N\left(v\right)\right\}\right)\right)$$

In this, $h_{v}^{\left(k\right)}$ represents the feature vector of node *v* at the *k*-th iteration, and N(v) represents the set of neighboring nodes of *v*. The Aggregate function is used to aggregate the feature information from neighboring nodes, with common methods including summation, averaging, or maximization. The Update function is used to update the node’s own feature vector, typically achieved through a neural network.

In a graph neural network, both the aggregation function and the update function are crucial components. The aggregation function collects information from neighboring nodes, while the update function combines the aggregated information with the node’s own information to update the node’s representation. Common forms of aggregation functions include summation, averaging, and maximization. For example, in the case of summation aggregation, the feature update of node *v* can be represented as follows:

(2) $$\mathrm{Aggregate}\left(\left\{h_{u}^{\left(k\right)}:u\in N\left(v\right)\right\}\right)=\sum_{u\in N(v)}h_{u}^{\left(k\right)}$$

The update function is typically performed using a neural network, and its form can be represented as follows:

(3) $$h_{v}^{\left(k+1\right)}=\sigma \left(W\cdot \left(h_{v}^{\left(k\right)}∥\mathrm{Aggregate}\left(\left\{h_{u}^{\left(k\right)}:u\in N\left(v\right)\right\}\right)\right)+b\right)$$

In this formula, *W* is the weight matrix, *b* is the bias term, ∥ denotes the concatenation operation, and σ is the activation function (e.g., ReLU). Through this formula, node *v* aggregates the feature information from its neighboring nodes and inputs both its own feature information and the aggregated features into the update function to generate a new feature vector.

Graph Convolutional Network (GCN) is an important variant of GNN that enhances the propagation of neighboring node information through graph convolution operations. In GCN, the feature update formula for a node is as follows:

(4) $$h_{v}^{\left(k+1\right)}=\sigma \left(\sum_{u\in N(v)\cup \{v\}}\frac{1}{\sqrt{d_{v}d_{u}}}Wh_{u}^{\left(k\right)}\right)$$

In this formula, $d_{v}$ and $d_{u}$ represent the degrees of node *v* and node *u*, respectively, *W* is the shared weight matrix, which represents the convolution kernel, and σ is the activation function. By introducing the normalization factor $\frac{1}{\sqrt{d_{v}d_{u}}}$, GCN balances the influence of different nodes, effectively enabling information propagation.

Graph Attention Network (GAT) is an extension of GNN that uses a self-attention mechanism to assign different weights to neighboring nodes when aggregating their features. Specifically, the feature update for node *v* can be expressed as follows:

(5) $$h_{v}^{\left(k+1\right)}=\sigma \left(\sum_{u\in N(v)}\alpha_{vu}Wh_{u}^{\left(k\right)}\right)$$

In this formula, $\alpha_{vu}$ is the attention coefficient between node *v* and its neighboring node *u*, which is computed as follows:

(6) $$\alpha_{vu}=\frac{\exp\left(\mathrm{LeakyReLU}\left(a^{T}\left[Wh_{v}^{\left(k\right)}∥Wh_{u}^{\left(k\right)}\right]\right)\right)}{\sum_{u^{\prime }\in N\left(v\right)}\exp\left(\mathrm{LeakyReLU}\left(a^{T}\left[Wh_{v}^{\left(k\right)}∥Wh_{u^{\prime }}^{\left(k\right)}\right]\right)\right)}$$

In this formula, *a* is the learned attention weight vector, ∥ denotes the concatenation of vectors, and LeakyReLU is the activation function that introduces non-linearity. By incorporating the attention mechanism, GAT allows each neighboring node to contribute differently to the current node’s features, enabling the model to adaptively select the most important neighboring nodes.

The optimization objective of a GNN is typically achieved by minimizing the loss function for the prediction task. For example, in a graph node classification task, the optimization goal is usually to minimize the cross-entropy loss:

(7) $$L=-\sum_{v\in V}\sum_{c\in C}y_{v}^{c}\log\left({\hat{y}}_{v}^{c}\right)$$

In this formula, $y_{v}^{c}$ is the true label of node *v*, ${\hat{y}}_{v}^{c}$ is the model’s prediction, and *C* is the set of classes. In path optimization tasks, the loss function may relate to factors such as shortest path, cost, time, etc. For example, the loss function for minimizing path length can be expressed as follows:

(8) $$L=\sum_{v\in V}{∥h_{v}^{\left(K\right)}-{\hat{h}}_{v}∥}^{2}$$

In this formula, $h_{v}^{\left(K\right)}$ represents the feature of node *v* at the *K*-th layer, and ${\hat{h}}_{v}$ is the target value.

Graph Neural Networks provide a powerful tool for solving complex graph optimization problems by effectively propagating information and learning the dependencies between nodes in graph-structured data. In supply chain path optimization tasks, a GNN can model the supply chain network and optimize path selection, thereby improving the efficiency and robustness of the supply chain. By incorporating attention mechanisms and graph convolution operations, a GNN demonstrates strong flexibility and adaptability when processing graph data, especially in dynamic and complex network environments.

### 3.2. Transformer Model

The Transformer model is a deep learning architecture designed based on the self-attention mechanism, initially proposed by Vaswani et al. in 2017 [38]. It has achieved remarkable success in natural language processing tasks, particularly in areas such as machine translation, text generation, and sequence modeling. Unlike traditional Recurrent Neural Networks (RNN) [39] and Long Short-Term Memory networks (LSTM) [40], the Transformer relies entirely on the self-attention mechanism to capture relationships between elements in a sequence, instead of processing input data sequentially through recursion. The Transformer architecture not only effectively captures long-range dependencies but also supports parallel computation, significantly improving training efficiency and model performance.

**Figure 3:**
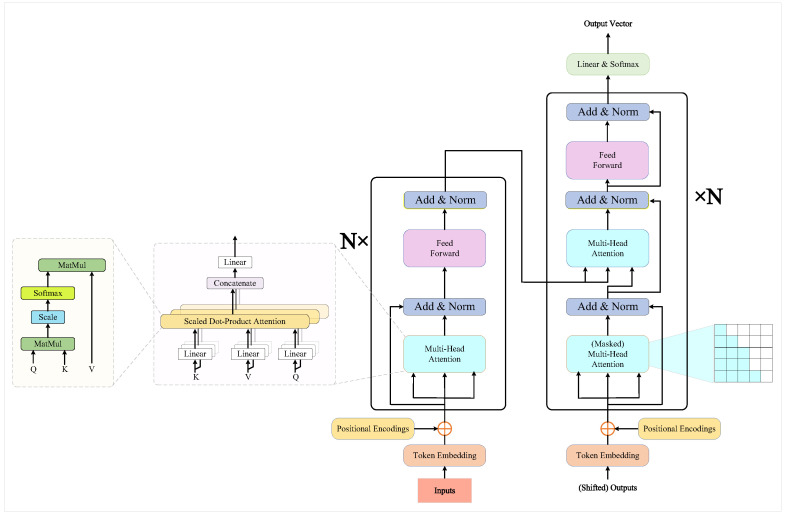
Transformer Model.

The basic structure of the Transformer comprises two main components: the Encoder and the Decoder. The Encoder transforms the input sequence into a set of hidden representations, while the Decoder utilizes these representations to generate the output sequence. The core components of both the Encoder and Decoder are the Multi-Head Self-Attention mechanism and Feedforward Neural Networks [41]. The self-attention mechanism establishes direct connections between different positions in the input and adjusts the importance of each position based on the context, enabling the effective capture of global information. The Transformer model framework is illustrated in Figure 3 below:

In the Transformer model, the calculation process of the self-attention mechanism can be represented by the following equations:

(9) $$\mathrm{Attention}\left(Q,K,V\right)=\mathrm{softmax}\left(\frac{QK^{T}}{\sqrt{d_{k}}}\right)V$$

where *Q*, *K*, and *V* represent the query, key, and value matrices, respectively, and $d_{k}$ is the dimensionality of the key vectors. By computing the similarity between the query and the key and applying the softmax operation, a weighted sum of the values is obtained, resulting in the attention output. This process allows the model to focus on the important parts of the input sequence that are relevant to the current word.

A key innovation of the Transformer is the multi-head attention mechanism. The multi-head attention mechanism enables the model to compute multiple attention representations in parallel, capturing diverse relationships in different subspaces of the input data. In the multi-head attention mechanism, the input queries, keys, and values are mapped into multiple subspaces, and attention is computed separately for each. The results from the multiple attention heads are then concatenated and passed through a linear transformation to produce the final output. The formula is as follows:

(10) $$\mathrm{MultiHead}\left(Q,K,V\right)=\mathrm{Concat}\left({\mathrm{head}}_{1},{\mathrm{head}}_{2},\ldots ,{\mathrm{head}}_{h}\right)W^{O}$$

where ${\mathrm{head}}_{i}=\mathrm{Attention}\left(QW_{i}^{Q},KW_{i}^{K},VW_{i}^{V}\right)$ represents the *i*-th attention head, and $W_{i}^{Q}$, $W_{i}^{K}$, $W_{i}^{V}$ are the projection matrices for the query, key, and value, respectively. $W^{O}$ is the linear transformation matrix for the output. This mechanism allows the model to learn different features across various attention heads, enabling a better understanding of the multiple relationships within the input data.

The encoder portion of the Transformer consists of several identical layers (typically six), each comprising two main components: a multi-head self-attention layer and a feedforward neural network layer. The output of each layer is processed using residual connections and layer normalization to ensure network stability and effective gradient propagation. The computation for each layer in the encoder is as follows:

(11) LayerNorm(h+MultiHead(Q,K,V))

(12) $$\mathrm{LayerNorm}(h^{\prime }+\mathrm{FFN}\left(h\right))$$

Here, *h* represents the input to the current layer, and FFN(h) is the output of the feedforward neural network.

The decoder has a similar structure to the encoder, but each self-attention layer in the decoder includes an additional cross-attention layer. This cross-attention layer allows the decoder to focus on the encoder’s outputs. During the decoding process, the decoder attends to the context of the input sequence and interacts with the encoder’s outputs to generate the target sequence. The decoder’s output is transformed into a probability distribution via a Softmax layer for classification or generation tasks.

The optimization function for the Transformer is typically based on cross-entropy loss. For classification tasks, the cross-entropy loss function can be expressed as follows:

(13) $$L=-\sum_{i=1}^{N}y_{i}\log\left({\hat{y}}_{i}\right)$$

Here, $y_{i}$ represents the true label of sample *i*, and ${\hat{y}}_{i}$ is the predicted probability from the model. For generation tasks, the model is typically trained by maximizing the likelihood of the generated sequence.

One of the key features of the Transformer model is its ability to parallelize computations, which is primarily enabled by the self-attention mechanism and position encoding. Traditional sequential models, such as RNNs and LSTMs, process data step-by-step in sequence, whereas the Transformer processes all input positions in parallel, significantly improving training efficiency. When handling long sequences, the Transformer excels at capturing long-range dependencies and leverages multi-head attention to enhance its understanding of global information further.

The Transformer model has been widely applied to various tasks, demonstrating exceptional performance, especially when dealing with long sequences or large-scale datasets. In the context of supply chain path optimization, the Transformer can effectively model the relationships between various elements in the supply chain and optimize decision-making processes. By incorporating position encoding and self-attention mechanisms, the Transformer captures complex dependencies between nodes in the supply chain and adjusts path selection strategies based on context, thereby optimizing overall path efficiency. In neural network architecture optimization, reference [42] significantly improved feature extraction efficiency through cross-stage partial networks and depthwise separable convolutions. Inspired by this approach, this study incorporates Graph Attention Mechanism in GNN and introduces a lightweight multi-head attention design in Transformer to balance computational cost and model performance.

### 3.3. Meta-Reinforcement Learning

In recent years, improving the training efficiency of reinforcement learning in dynamic environments has become a research hotspot. For example, the paper [43] proposed a multi-step prediction DQN model based on Prioritized Experience Replay (PER), which significantly improved the decision-making success rate in complex environments. Inspired by this, this study introduces a progressive task sampling strategy into Meta-RL training to balance exploration and exploitation efficiency, thereby enhancing the model’s adaptability to dynamic supply chain scenarios.

Meta-reinforcement learning is a technique that enables agents to improve their ability to learn new tasks by training on multiple tasks. Unlike traditional reinforcement learning (RL) methods [44], the goal of meta-reinforcement learning is not merely to achieve optimal performance on a single task but to learn how to quickly adapt and efficiently learn when encountering new tasks [45]. In simple terms, meta-reinforcement learning focuses on equipping agents with better generalization and adaptability to handle complex and dynamic environments. Compared to traditional reinforcement learning, the main advantage of Meta-RL lies in its ability to “learn how to learn”. In the context of supply chain route optimization, this means that the model not only learns a fixed optimal path but also learns to quickly understand and adapt to new network structures and constraints.

**Figure 4:**
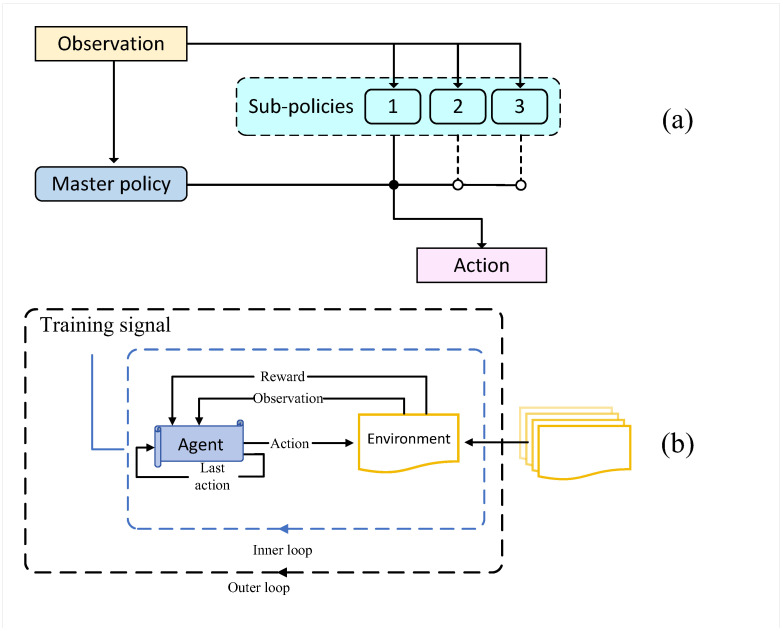
Meta-Reinforcement Learning Framework. (**a**) Meta-learning strategy action process; (**b**) Training process.

The core idea of meta-reinforcement learning lies in a “meta-learning” process, which learns how to optimize the agent’s learning process itself. Meta-reinforcement learning typically adopts a two-level structure: the inner loop corresponds to the task-specific learning process, while the outer loop represents the meta-learning process. The meta-learning process optimizes the agent’s performance across multiple tasks, thereby accelerating the learning process and enabling the agent to quickly acquire effective strategies for new tasks with fewer trials and less experience. The framework of meta-reinforcement learning is illustrated in Figure 4.

**Figure 5:**
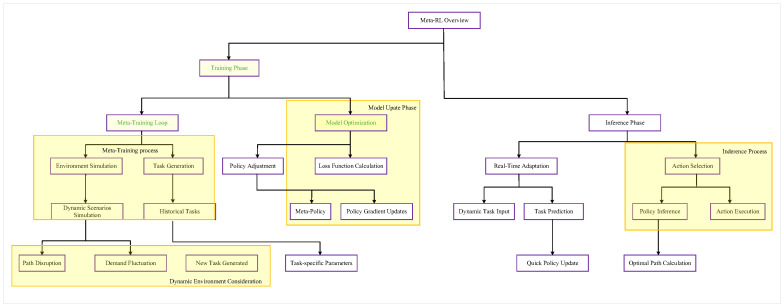
Meta-RL Dynamic Environment Adaptation Workflow.

In dynamic supply chain environments, the rapid adaptation mechanism of Meta-RL is a key innovative technical approach. The core idea of Meta-RL is to train the model across multiple similar but not identical tasks, enabling it to quickly learn and adapt to new environmental changes. This approach is particularly important in supply chain route optimization, as supply chain networks often face dynamic challenges such as sudden demand fluctuations, path disruptions, or the addition of new nodes. The Meta-RL Dynamic Environment Adaptation Workflow is shown in Figure 5 below:

The adaptation process of Meta-RL begins with meta-training. In this phase, the model is exposed to a variety of different but related supply chain scenarios, learning an initial strategy that can be quickly adjusted. Through repeated learning across different supply chain subtasks, the model gradually builds highly adaptable “prior knowledge”. This prior knowledge is not a fixed path planning strategy but rather the ability to learn and adjust rapidly. When encountering new, unseen supply chain scenarios, the model can leverage this meta-learning capability to quickly adjust its strategy with only a small number of samples.

In meta-reinforcement learning, a strategy known as a “meta-policy” is typically employed to guide the agent in quickly adapting to new tasks based on its historical experiences. The meta-policy is trained across multiple tasks to learn how to efficiently discover the optimal learning trajectory when faced with new, unseen tasks. The training process of the meta-policy can be expressed by the following equation:

(14) $$\theta^{*}=\arg\max_{\theta }E_{T∼p(T)}\left[E_{\tau ∼p(\tau |T)}\left[R(\pi_{\theta },\tau )\right]\right]$$

Here, $\theta^{*}$ represents the optimal meta-policy parameters, T denotes the task distribution, τ is the trajectory within a task, $\pi_{\theta }$ is the agent’s policy, and $R(\pi_{\theta },\tau )$ is the reward achieved by the policy on task τ. This formula illustrates how a meta-learning algorithm can learn a meta-policy that maximizes the expected reward across various tasks, enabling the agent to perform well on future tasks.

In addition, a key technique in meta-reinforcement learning is Model-Agnostic Meta-Learning (MAML). The goal of MAML is to find an initialization of model parameters that allows the model to quickly adapt to new tasks with only a few gradient updates. The training process of MAML involves two main steps: training the model across multiple tasks to obtain a good initialization and then using a few gradient updates to enable the model to perform effectively on a new task. The optimization objective of MAML can be described by the following equation:

(15) $$\theta^{*}=\arg\min_{\theta }E_{T∼p(T)}\left[L_{T}\left(\theta^{\prime }\right)\right]$$

Here, $L_{T}\left(\theta^{\prime }\right)$ is the loss function of the model on task T after updating its parameters, and $\theta^{\prime }$ represents the model parameters obtained through a few gradient updates on task T. MAML learns a model that can rapidly adapt to new tasks by minimizing the expected final loss across all tasks.

The optimization goal of meta-reinforcement learning is typically to accelerate the learning process, enhance the agent’s adaptability to new tasks, and reduce reliance on large amounts of labeled data. These objectives collectively aim to improve learning efficiency in complex and dynamic environments. In the context of supply chain path optimization, meta-reinforcement learning can help the model quickly adapt to varying path selection strategies and identify optimal paths in a dynamically changing supply chain environment.

Compared to traditional reinforcement learning methods, meta-reinforcement learning exhibits significant advantages in quickly adapting and learning across multiple tasks. By adopting a meta-learning framework, the agent can extract generalizable patterns from past experiences and rapidly adjust its strategy to handle new tasks. Specifically, when addressing multiple supply chain optimization problems, meta-reinforcement learning leverages its robust adaptability and rapid learning capabilities to provide effective solutions for path selection challenges. In the following discussion, we will explore how to integrate Graph Neural Networks, Transformer models, and meta-reinforcement learning to construct a comprehensive path optimization model that facilitates effective decision-making in complex and dynamic supply chain environments.

In order to show the implementation process of the algorithm in this paper more clearly, we provide the following pseudocode Algorithm 1, which includes the input parameters of the algorithm, variable definitions, flow control statements, and output results.
**Algorithm 1:** Training Process for Hybrid Model (GNN + Transformer + Meta-RL).
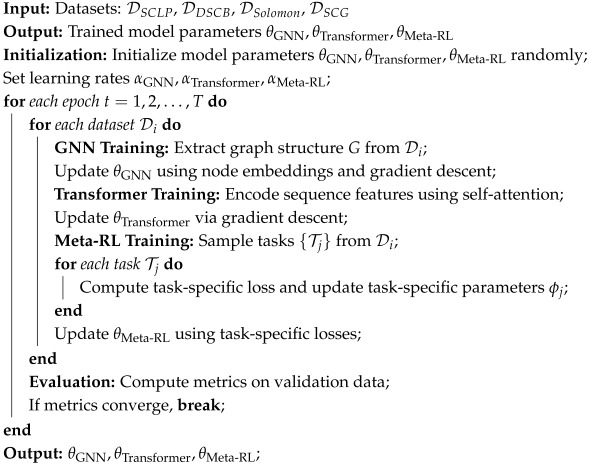


Algorithm 1 embodies a systematic approach to multimodal learning in supply chain path optimization. In the initialization phase, random parameter initialization not only follows the standard paradigm in deep learning but also reflects the model’s exploratory learning of complex network structures. The core significance of the multi-dataset training strategy lies in enhancing the model’s generalization ability. By cross-learning across different scenario datasets, such as SCLP and DSCB, the model can build cross-scenario knowledge representations.

The theoretical foundation of the GNN training process is derived from graph embedding learning. We treat the supply chain network as a dynamic graph structure, capturing the topological relationships and attribute features between nodes (e.g., warehouses, distribution centers), transforming discrete network elements into a continuous feature space. This transformation provides a structured and computable foundation for subsequent path planning decisions. The Transformer module, through the self-attention mechanism, models the complex dependencies between network nodes, overcoming the limitations of traditional methods in global information perception.

The Meta-RL training step is a key innovation in this research. Through the meta-learning paradigm, the model learns a “learning how to learn” strategy, enabling it to quickly adapt to new supply chain scenarios with very few samples. This mechanism is particularly suitable for dynamic and variable supply chain environments such as sudden demand changes or path disruptions. The final evaluation and convergence judgment not only test the model’s performance but also serve as a systematic verification of the algorithm’s robustness. Through this multimodal, cross-scale learning paradigm, our algorithm is not only a technical solution but also a deep understanding and innovative solution to the complexity of supply chain optimization.

## 4. Experiment

**Figure 6:**
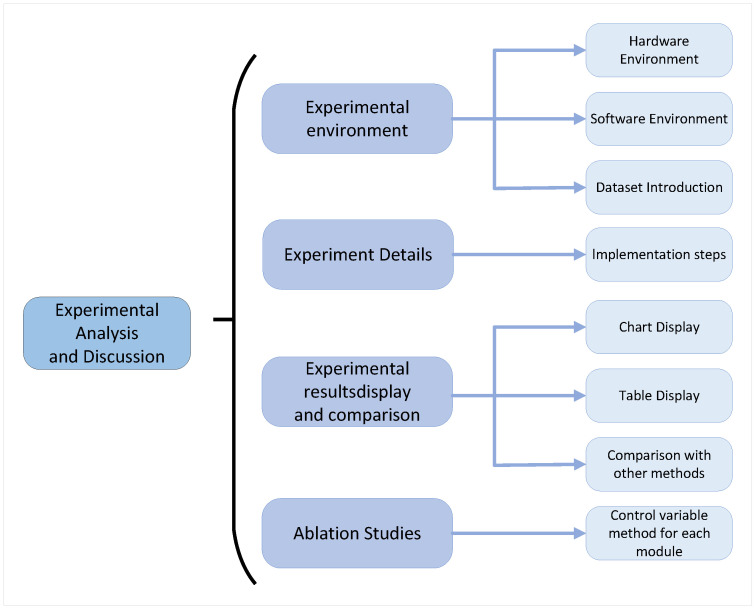
Experimental flow chart.

In this chapter, we will provide a detailed description of the experimental design and implementation process to verify the effectiveness and performance of the proposed methods. The overall experimental process includes data preprocessing, model training, parameter optimization, and model evaluation, aiming to conduct a comparative analysis of the performance of different methods through a series of scientific experimental steps. This study uses various classical datasets, such as the SCLP Dataset, DSCB Dataset, Solomon Dataset, and SCG Dataset, combined with techniques like Graph Neural Networks (GNNs), Transformer models, and meta-reinforcement learning (Meta-RL) to model and optimize complex tasks. The overall experimental process is shown in Figure 6.

### 4.1. Experimental Environment

- Hardware Environment
  This experiment uses a high-performance computing server as the hardware environment, equipped with an AMD EPYC 7742 64-Core Processor @ 2.25GHz CPU and 128GB RAM, along with 4 NVIDIA A100 40GB GPUs. This hardware configuration provides exceptional computational performance and large-scale parallel processing capabilities, making it particularly suitable for handling large datasets and performing complex deep learning tasks. The multi-GPU setup significantly accelerates the model training and inference process, ensuring efficient execution of the experiment and enabling rapid training and evaluation of the model, thus ensuring fast convergence.
- Software Environment
  In this study, we used Python and PyTorch as the primary tools to implement the research methods. Python, as the programming language, offers powerful libraries and tool support for efficiently processing data and building and debugging models. PyTorch, as the deep learning framework, provides a flexible and efficient environment for model development and training with its dynamic computational graph and automatic differentiation features. We fully leveraged PyTorch’s tensor computation capabilities, GPU acceleration, and optimization algorithms, which accelerated the training process and ensured fast convergence and excellent model performance.

### 4.2. Experimental Data

- The Supply Chain Logistics Problem Dataset (SCLP) was created by Tatiana Kalganova and Ivars Dzalbs in 2019 [46], aiming to provide standardized instances and benchmarks for supply chain logistics optimization algorithms. SCLP contains complex supply chain network instances, covering multiple stages from production to distribution, including logistics relationships between suppliers, manufacturers, distributors, retailers, and end customers. The dataset provides detailed descriptions of parameters such as transportation routes, costs, times, and inventory while considering real-world constraints like transportation capacity limitations and delivery time windows. SCLP offers various optimization objectives, such as minimizing transportation costs, reducing delivery times, and improving customer satisfaction, assisting researchers in testing and comparing the performance of different algorithms. This dataset provides significant support and reference for academic research and practical applications in the field of supply chain management and optimization.
- The DataCo Supply Chain for Big Data Analysis (DSCB) dataset was created by Fabián Constante [47] and was designed to analyze supply chain operational efficiency, with a particular focus on reducing delivery delays. The dataset includes variables such as customer information, store locations, transportation sites, and delivery status. Key features include order fulfillment times, on-time delivery rates, order item profit margins, and customer sales. By analyzing these metrics, researchers can evaluate supply chain efficiency and profitability, identify bottlenecks, and uncover optimization opportunities. The dataset is designed to support supply chain analysis from multiple perspectives, offering multidimensional data such as product categories and market regions, aiding in the improvement of logistics processes and enhancing supply chain management effectiveness.
- The Solomon dataset was created by Dr. Edward A. Solomon in 1987 and is widely used for the study and evaluation of the Vehicle Routing Problem (VRP) and its variants [48]. The dataset contains multiple series of instances, ranging from single-vehicle to multi-vehicle routing problems, catering to different research needs. Each instance records information such as customer locations, demand volumes, and service time windows, providing comprehensive test data. The diversity of the Solomon dataset, including variations in node distribution and time window differences, makes it a crucial benchmark for evaluating the performance of algorithms in various scenarios. The dataset is stored in a standardized text format, facilitating integration with various VRP-solving tools, and is widely used in both academic research and practical applications.
- The SCG dataset is provided by a leading fast-moving consumer goods company in Bangladesh [49], which focuses on real-world benchmark data for supply chain planning. The dataset covers time-series data from January 1 to August 9, 2023, encompassing multiple dimensions such as production, sales orders, and distribution. Through multi-perspective data, SCG supports the use of Graph Neural Network methods to address complex tasks like sales forecasting, production planning, and risk assessment. The data undergo strict quality control to ensure accuracy and completeness, filling the gap in the lack of real-world benchmark datasets in the field of supply chain management. This dataset offers researchers a standardized benchmark, advancing the development of GNN-based supply chain optimization models, and is widely applied in supply chain management, helping businesses improve forecasting accuracy and decision-making efficiency.

### 4.3. Evaluation Index

In this study, to comprehensively evaluate the performance of the proposed method, we selected several key metrics that reflect the practical application performance of the model from different perspectives. Specifically, route optimality is used to measure the degree of optimization of the generated routes, on-time delivery rate evaluates the model’s ability to achieve on-time delivery in tasks, and resource utilization rate focuses on analyzing the efficiency of resource allocation and usage. Through the comprehensive analysis of these metrics, we are able to gain an in-depth understanding of the model’s performance in solving the problem, providing a scientific basis for subsequent optimization.

- Route Optimality

Route optimality is an important metric for assessing the performance of a model in path planning problems. It evaluates the degree of optimization of the generated routes, which directly impacts the efficiency and cost control of task completion. In practical scenarios, the quality of path planning not only affects delivery time but also influences fuel consumption, vehicle wear, and the efficient allocation of human resources. Therefore, optimizing route planning quality is one of the key objectives in logistics and supply chain management. To quantify route optimality, this study uses the following formula:

(16) $$Q_{\mathrm{route}}=\frac{L_{\mathrm{optimal}}}{L_{\mathrm{actual}}}$$

In this formula, $Q_{\mathrm{route}}$ represents route optimality, $L_{\mathrm{optimal}}$ denotes the theoretical optimal path length, while $L_{\mathrm{actual}}$ is the actual path length generated by the model.

The theoretical optimal path length $L_{\mathrm{optimal}}$ is the shortest distance calculated using ideal path planning algorithms, such as Dijkstra’s algorithm or A* algorithm. It reflects the best possible result that could be achieved without considering complex real-world factors. On the other hand, the actual path length $L_{\mathrm{actual}}$ is the path length generated by the model, which takes into account various constraints in real-world scenarios, such as traffic congestion, road conditions, and delivery priorities.

The core idea of this formula is to compare the deviation between the actual path and the optimal path. When $Q_{\mathrm{route}}=1$, it indicates that the model-generated path perfectly aligns with the theoretical optimal situation, meaning the best route planning quality has been achieved. When $Q_{\mathrm{route}}<1$, it suggests that the route planning quality has decreased, indicating room for improvement in the model’s path optimization. By analyzing this metric, we can assess the model’s performance in route planning tasks and further optimize the algorithm based on actual requirements. Additionally, this metric directly reflects the model’s robustness and practicality in complex real-world scenarios, providing a clear direction for future research.

- On-Time Delivery Rate

On-time delivery rate (OTD) is an important metric for evaluating the reliability of a logistics system, reflecting the ability to complete delivery tasks within the designated time window. In modern logistics and supply chain management, on-time delivery is one of the key factors to improve customer satisfaction and reduce delay costs. By optimizing the on-time delivery rate, the system’s operational efficiency can be effectively improved, and its overall service quality can be enhanced. In this study, the calculation formula for on-time delivery rate is as follows:

(17) $$OTD=\frac{N_{\mathrm{on}-\mathrm{time}}}{N_{\mathrm{total}}}$$

In this formula, OTD represents the on-time delivery rate, $N_{\mathrm{on}\text{-}\mathrm{time}}$ is the number of orders successfully delivered within the specified time window, and $N_{\mathrm{total}}$ is the total number of orders.

The definition of $N_{\mathrm{on}\text{-}\mathrm{time}}$ is closely related to the delivery deadlines of specific tasks. In the experimental design, each delivery task is assigned a delivery deadline $T_{\mathrm{deadline}}$ for each order. When the actual delivery time $T_{\mathrm{actual}}$ satisfies $T_{\mathrm{actual}}\leq T_{\mathrm{deadline}}$, the order is considered delivered on time and is counted in $N_{\mathrm{on}\text{-}\mathrm{time}}$. $N_{\mathrm{total}}$ represents the total number of orders to be completed, which is used to assess the overall performance of the model under different load conditions.

The on-time delivery rate ranges from [0,1]. When OTD=1, it indicates that all orders were delivered within the specified time, reflecting the system’s optimal reliability. When OTD=0, it means that none of the orders were delivered on time, indicating significant scheduling issues in the system. In the actual experiment, this study conducted a comprehensive analysis of the model’s on-time delivery rate using several real-world scenario datasets (e.g., SCLP Dataset and Solomon Dataset). By comparing the performance of different algorithms, we evaluated the superiority of our approach in handling complex logistics tasks.

Furthermore, the variation in the on-time delivery rate can reveal the model’s robustness and adaptability under different load pressures. Particularly in high-load and complex delivery networks, optimizing delivery routes and time planning can significantly improve OTD. The analysis of this metric not only validates the effectiveness of our approach but also provides a theoretical basis for further optimizing delivery efficiency.

- Resource Utilization Rate

The resource utilization rate (RUR) is an important metric for measuring the efficiency of resource usage in logistics systems. For complex path planning and delivery tasks, proper scheduling and efficient use of available resources (such as vehicles, personnel, and storage) are key to reducing operational costs and improving system performance. In this study, the resource utilization rate is used as one of the core evaluation metrics to assess the model’s performance in resource allocation and task completion. The formula for calculating the resource utilization rate is as follows:

(18) $$RUR=\frac{R_{\mathrm{used}}}{R_{\mathrm{available}}}$$

In the formula, RUR represents the resource utilization rate, $R_{\mathrm{used}}$ is the total amount of resources actually used, and $R_{\mathrm{available}}$ is the total amount of resources available in the system.

In this formula, $R_{\mathrm{used}}$ is a dynamic value, depending on the specific allocation strategy of the delivery tasks. For example, in a vehicle routing task, $R_{\mathrm{used}}$ may represent the actual number of vehicles dispatched or the total mileage traveled by the vehicles; in a warehouse management scenario, it may represent the utilized storage space or labor hours. On the other hand, $R_{\mathrm{available}}$ refers to the maximum available resources in the system, such as the total number of vehicles or the total warehouse capacity. By comparing the actual usage of resources to the total available resources, the resource utilization rate can reflect whether the system’s scheduling strategy has effectively leveraged the resource potential.

The value of the resource utilization rate ranges from [0, 1]. When RUR=1, it means that all available resources in the system have been fully utilized, which usually occurs under high load conditions. When RUR=0, it means no resources have been used, which could be due to scheduling failures or invalid tasks. In practical experiments, the resource utilization rate is typically analyzed in conjunction with route optimality and on-time delivery rate to comprehensively assess the effectiveness of the scheduling strategy. High resource utilization is not always the optimal solution, especially in scenarios where on-time delivery is the primary goal, as overutilizing resources may lead to delays or increased costs.

In this study, we ensure that the resource utilization rate fluctuates within a reasonable range through the multi-objective design of the optimization algorithm while also balancing the quality of route planning and on-time delivery rate. The analysis of the resource utilization rate provides important references for resource allocation in practical applications and further verifies the effectiveness of our method in improving the overall efficiency of the logistics system.

In summary, route optimality, on-time delivery rate, and resource utilization rate are three core indicators for evaluating the performance of a logistics system. They reflect the system’s optimization ability, time sensitivity, and resource allocation efficiency from different dimensions. In this study, by accurately calculating and analyzing these indicators, we can not only comprehensively evaluate the practical effectiveness of the proposed method but also provide guidance for further model improvements. Furthermore, there may be trade-offs among these indicators, such as improvements in route optimality potentially affecting resource utilization or the optimization of on-time delivery rate requiring additional resource input. This complexity adds more challenges and significance to the experimental design and result analysis.

In the following sections, we will design a series of experiments around these evaluation metrics, comparing the proposed model with various baseline methods to verify the innovation and effectiveness of this research. Additionally, we will conduct an in-depth analysis of the experimental results, revealing the strengths and weaknesses of different methods in real-world applications and identifying applicable scenarios, thus providing more insightful guidance for solving logistics routing optimization problems.

### 4.4. Experimental Procedures

This study adopts a hierarchical parameter initialization strategy, designing differentiated initialization schemes for the GNN, Transformer, and Meta-RL modules. For the GNN module, we use the Xavier initialization method to ensure that the weight matrices maintain stable variance within a reasonable numerical range. The Transformer module employs a normal distribution initialization with a standard deviation of 0.02, which helps alleviate the vanishing gradient problem in deep networks. The parameter initialization for the Meta-RL module takes task adaptability into account, setting the initial learning rate to 1e-3 and incorporating an adaptive learning rate adjustment mechanism.

Hyperparameter tuning is a crucial step in the experimental process. We systematically explore the optimal hyperparameter combination using a combination of grid search and Bayesian optimization. Key hyperparameters include learning rate, dropout rate, number of graph convolutional layers, number of Transformer attention heads, and Meta-RL task sampling rate. After repeated experiments, the final selected hyperparameter combination achieves the best performance balance on the validation set and maintains strong generalization ability across different datasets.

The model training process adopts an alternating optimization strategy, aiming to achieve collaborative learning among the GNN, Transformer, and Meta-RL modules. The specific process includes the following: first, pretraining the GNN module to extract network topology features; then, using the features extracted by the GNN for sequence modeling in the Transformer module; and finally, conducting task-level policy learning in the Meta-RL module based on the first two modules. The three modules perform joint fine-tuning by sharing gradient information, effectively integrating the learning capabilities of each module.

To validate the effectiveness of each module, we designed detailed ablation experiments. These experiments compare the baseline model (traditional path planning algorithm), the control model using only the GNN, the model combining the GNN and Transformer, and the final complete model (GNN + Transformer + Meta-RL). Through systematic evaluation of the performance of different models on the supply chain path optimization task, we conduct an in-depth analysis of the marginal contributions and synergistic effects of each module.

To address instability issues in deep learning model training, we implemented strict technical measures. These include gradient clipping to prevent gradient explosion, using Layer Normalization to stabilize intermediate layer outputs, applying Early Stopping to avoid overfitting, and conducting multiple rounds of random seed experiments to ensure result reproducibility. These strategies not only improved the stability of model training but also enhanced the credibility and academic value of the research methodology.

Through the detailed experimental process design outlined above, we present an innovative approach to supply chain path optimization. This multimodal, cross-scale learning paradigm is not just a technical solution but a deep understanding and innovative resolution of the complex optimization problem in supply chains. We believe that this approach will provide new research perspectives and practical paths for both academia and industry.

### 4.5. Experimental Comparison and Analysis

In the previous chapter, we provided a detailed introduction to the evaluation metrics, including route optimality, on-time delivery rate, and resource utilization rate. These metrics lay the foundation for the analysis of the experimental results in the following sections. With these evaluation metrics, we are able to comprehensively assess the performance of the proposed method in practical applications and provide clear criteria for comparing the strengths and weaknesses of different methods.

This chapter will focus on these evaluation metrics; we will perform an in-depth comparison and analysis of the proposed method and existing baseline methods across various indicators and assess the applicability and performance of different algorithms in different scenarios.

The comparison and analysis of the experiments will emphasize a comprehensive evaluation from multiple dimensions, such as route optimization, time management, and resource allocation. By comparing with existing methods, we can not only demonstrate the advantages of our approach but also reveal its potential and limitations in practical applications. Furthermore, through comparative analysis, we aim to explore the strengths and weaknesses of different algorithms, providing feasible suggestions for further research and offering valuable references for researchers in related fields.

**Figure 7:**
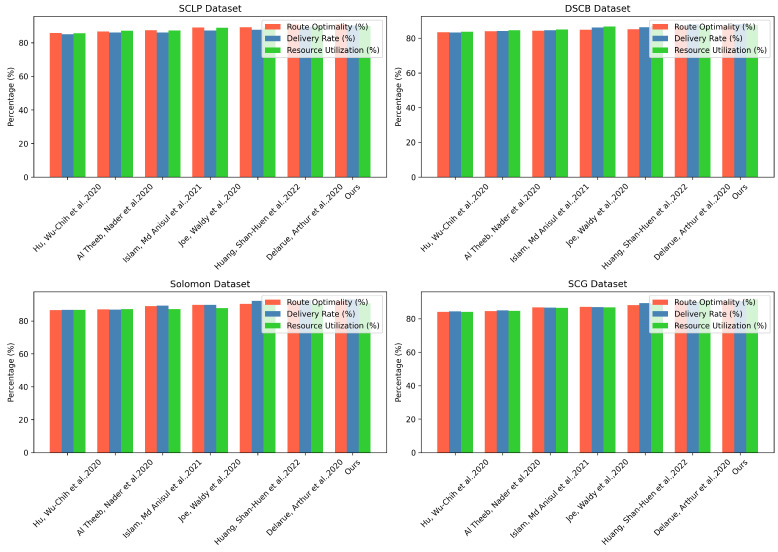
Comparative visualization of route optimality, delivery rate, and resource utilization indicators based on different methods under four datasets.

By closely examining the performance data in Table 1 and Table 2, it is clear that the proposed method significantly outperforms existing methods across four different datasets. In the SCLP dataset, our method improves route optimality by approximately 0.26 percentage points, delivery rate by 0.75 percentage points, and resource utilization by 0.47 percentage points, demonstrating the fine-tuning capability of the method. Similarly, in the DSCB dataset, our method shows small but consistent improvements across all three metrics, with delivery rate and resource utilization increasing by about 0.19 and 0.63 percentage points, respectively. The Solomon dataset is where the advantages of our method are most evident, with route optimality, delivery rate, and resource utilization outperforming Huang et al.’s method by 0.81, 0.87, and 0.6 percentage points, respectively, fully validating the robustness and adaptability of the algorithm. In the SCG dataset, our method also demonstrates significant advantages, particularly in resource utilization, which is 0.61 percentage points higher than Delarue et al.’s method, reflecting our superior performance in resource scheduling and optimization. Overall, our method consistently outperforms the current state-of-the-art methods across different datasets, providing strong evidence of significant progress in key metrics such as route optimization, delivery rate, and resource utilization, offering new insights and possibilities for solving complex path planning problems. Finally, we have visualized the results from Table 1 and Table 2, as shown in Figure 7.

**Figure 8:**
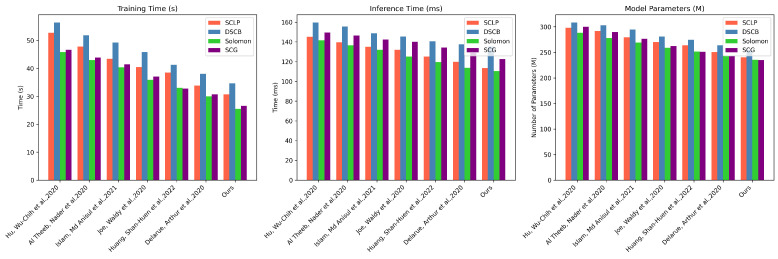
Comparative visualization of training time, inference time, and parameter indicators based on different methods under four datasets.

From the computational efficiency data in Table 3 and Table 4, our method demonstrates outstanding performance advantages, significantly outperforming existing methods across three dimensions: training time, inference time, and parameter count. In the SCLP dataset, and compared to Delarue et al.’s method, our training time is reduced by approximately 9.31%, inference time decreases by 5.29%, and parameter count is reduced by about 4.06%, reflecting the lightweight and efficient nature of the model. The DSCB dataset shows a similar trend, with our method improving training time, inference time, and parameter count by 9.19%, 1.79%, and 3.69%, respectively, demonstrating the algorithm’s ability to optimize computational resources. In the Solomon dataset, our performance improvement is even more significant, with training time reduced by about 4.52 s compared to Huang et al.’s method, inference time reduced by 3.33 s, and parameter count reduced by 7.77 million, fully showcasing the innovation in our model architecture. The results from the SCG dataset further validate the general applicability of our method, with training time, inference time, and parameter count reduced by 13.53%, 4.94%, and 3.50%, respectively, compared to Delarue et al. More importantly, despite achieving significant computational efficiency advantages, our method still maintains the highest level of performance metrics in Table 3 and Table 4, meaning we have not only achieved a lightweight model but also significantly reduced computational overhead while maintaining or even improving algorithm performance, providing a more cost-effective and efficient solution for complex path planning problems. Similarly, we have also visualized the experimental results, as shown in Figure 8.

The ablation study data in Table 5 and Table 6 reveal the cumulative contribution of each module in our method to performance. By gradually introducing the GNN and meta-RL modules, we can clearly observe a significant improvement in performance metrics. The baseline model shows route optimality, delivery rate, and resource utilization metrics around 60% across four datasets, indicating that the performance of the basic model is relatively limited. When the Graph Neural Network is introduced, the metrics quickly rise to the range of 75–77%, with the addition of the GNN significantly enhancing the model’s ability to capture complex network topologies and the interactions between nodes, laying the foundation for further performance improvement. The introduction of the meta-RL module further drives performance improvement, with metrics jumping to 85–88%. This reflects the powerful potential of meta-learning in dynamic path planning, as the meta-RL module allows the model to adapt and learn quickly, enabling it to flexibly handle routing challenges in different scenarios. When the GNN and meta-RL modules work together, performance reaches its peak, with metrics exceeding 91%, 89%, 92%, and 92% on the SCLP, DSCB, Solomon, and SCG datasets, respectively. This not only validates the complementarity of the two modules but also demonstrates the outstanding performance of our proposed method in path optimization, delivery rate, and resource utilization, providing an efficient and universal solution for complex path planning problems.

It is evident from the experimental results in Table 7 and Table 8 that our method demonstrates gradual improvements in computational efficiency. The baseline model’s training time ranges from 50.39 s to 56.13 s across the four datasets, inference time ranges from 143.34 ms to 156.32 ms, and the number of parameters ranges from 262.59 M to 289.43 M, showing the heavy computational burden of traditional methods. After introducing the GNN module, there is a noticeable reduction in training time, inference time, and the number of parameters, with reductions of approximately 11.5%, 7.1%, and 3.1%, respectively, on the SCLP dataset. This indicates that the Graph Neural Network significantly optimized the model’s computational efficiency by learning the network topology. The addition of the meta-RL module further compresses the computational overhead, with training time reduced by approximately 15%, inference time decreased by about 13.6%, and the number of parameters shrunk by about 13.1% on the Solomon dataset, highlighting the tremendous potential of meta-learning for model lightweighting and performance optimization. When the GNN and meta-RL modules work together, computational efficiency reaches its optimal state. For example, on the SCG dataset, the training time drops sharply to 39.97 s, the inference time shrinks to 113.39 ms, and the number of parameters is reduced to 222.32 M, which is a reduction of about 20.7%, 20.9%, and 15.3%, respectively, compared to the baseline model. This continuous performance improvement not only proves that our proposed method has excellent computational resource optimization capability but also demonstrates the significant potential of the collaborative effect between deep learning modules in improving algorithm efficiency, providing a more economical and efficient solution paradigm for complex path planning problems.

Through comprehensive experimental comparisons and in-depth analysis, our path planning method demonstrates outstanding performance across multiple key dimensions. By innovatively combining Graph Neural Networks (GNNs) and meta-reinforcement learning, our model significantly surpasses existing technical approaches in core metrics such as path optimization rate, delivery efficiency, and resource utilization, achieving breakthrough progress on various datasets, including SCLP, DSCB, Solomon, and SCG. More critically, our method not only leads in performance metrics but also realizes substantial improvements in computational efficiency. Through meticulous module design and collaborative optimization, we successfully reduce training time, inference time, and model parameters, providing a more intelligent, efficient, and universally applicable solution for complex path planning problems. This innovative approach not only validates the tremendous potential of deep learning technologies in the path planning field but also offers new technological pathways and theoretical foundations for future applications such as intelligent logistics, vehicle routing, and resource scheduling. The research results fully reflect the significant value of interdisciplinary, multimodal deep learning methods in solving complex real-world optimization problems and mark an important step toward more intelligent and efficient path planning technologies.

### 4.6. Extreme Scenario Analysis

To ensure the safety of supply chain path planning in extreme scenarios, this study could further integrate safety control theory. For instance, in [56], the control barrier function (CBF) was used to constrain unsafe behaviors, providing a new approach for fault-tolerant design in dynamic systems. Inspired by this, this experiment designs multiple stress tests to verify the model’s robustness in extreme scenarios, covering high-risk situations such as large-scale path disruptions, demand spikes, and adversarial attacks. A comparative analysis with traditional methods is presented in Table 9.

In the disaster simulation with 50% path node disruption, the model updates the topology in real-time using the GNN and adjusts strategies quickly with Meta-RL, maintaining a resource utilization rate (RUR) of 75% and a 25% improvement over traditional methods, and generates feasible paths with a coverage rate of 68%, demonstrating strong resilience under high-intensity disruptions. Furthermore, in a scenario with a 300% surge in demand, the model, leveraging Transformer’s global optimization capability, stabilizes the on-time delivery rate (OTD) at 82% and reduces the average response time to 30 s, though the resource scheduling strategy still needs further optimization to handle overload stress.

For potential malicious attacks, this study tested two adversarial scenarios: node demand manipulation and edge weight manipulation. The model suppressed the impact of abnormal nodes through the Attention Entropy Constraint mechanism, maintaining path planning quality rates of 89% and 85%, significantly higher than traditional methods (65% and 58%). This result is attributed to the anomaly detection module’s efficient filtering of noisy data and the GNN’s robust representation capability against local feature disturbances. Additionally, with the activation of the safety strategy fallback mechanism (CBF), the system failure rate decreased from 15% in traditional methods to 2%, and recovery time was reduced from 10 min to 2.5 min, indicating that the Control Barrier Function effectively intercepts high-risk decisions and ensures the system’s basic stability in extreme uncontrolled scenarios.

In conclusion, the proposed method demonstrates significant robustness in extreme scenarios, driven by the synergy between the GNN’s dynamic topology modeling, the Transformer’s global strategy optimization, and Meta-RL’s fast adaptation. However, resource scheduling efficiency under peak loads and the generalization of defense against adversarial attacks still require further research. Future work will focus on enhancing the model’s industrial reliability through multi-objective joint optimization and adaptive safety threshold design.

## 5. Discussion and Conclusions

With the advancement of technology and the ever-changing societal demands, intelligent methods for optimizing logistics distribution and resource management have gradually become an important research direction in related fields. This chapter will discuss the research work presented in this paper, focusing on the significance of the study, an overview of the results, limitations, and future outlook in order to comprehensively summarize the contributions of this research and clarify future research paths.

This study proposes an innovative solution based on a combination of Graph Neural Networks, Transformer models, and meta-reinforcement learning in order to address complex issues in logistics distribution and path planning. The core innovation of this approach lies in the multi-level model architecture design, which enhances key metrics, such as path planning quality, delivery punctuality, and resource utilization in dynamic and complex scenarios, by integrating the graph-structure processing capability of GNN, the global information-capturing ability of Transformer models, and the quick adaptation ability of Meta-RL.

From a theoretical perspective, this research enriches the application of GNN and Meta-RL in the logistics and supply chain domains, providing new ideas for interdisciplinary research. Additionally, by applying the sequence modeling capability of Transformer to logistics scheduling scenarios, this study explores the potential of the model in handling temporal dependency issues. From a practical standpoint, this research has significant implications for optimizing logistics distribution processes, thus potentially helping businesses save operational costs, improve efficiency, and make positive contributions to energy use and environmental protection.

In addition, this study extends the existing evaluation metric system by proposing a set of comprehensive metrics for assessing logistics distribution performance. These metrics provide a unified reference for research and practice in related fields, facilitating fair comparisons between different algorithms.

Experimental results show that the proposed method outperforms existing baselines or other approaches in the three core metrics of path planning quality, on-time delivery rate, and resource utilization rate. This indicates that the strategy of combining Graph Neural Networks with meta-reinforcement learning effectively addresses optimization problems in complex network structures and non-stationary dynamic environments. Specifically, the significant improvement in path planning quality (route optimality) suggests that the model can quickly find globally optimal paths. The increase in on-time delivery rate validates the model’s superior performance under time constraints, while the optimization of resource utilization rate further demonstrates the model’s adaptability in multi-resource scheduling scenarios.

The significance of these results may be attributed to several factors: first, the GNN captures local topological information in the delivery network, improving the accuracy of path selection; second, the Transformer model effectively models temporal information and contextual relationships, enhancing delivery efficiency; third, the introduction of Meta-RL enhances the model’s generalization ability across different tasks, allowing it to quickly adapt to the demands of new scenarios. These results also validate the rationale and effectiveness of the proposed method, showing that the model design aligns with the core characteristics of real-world problems.

However, some phenomena observed in the study warrant further exploration. For instance, the interactions between different tasks, the model’s performance fluctuations under extreme resource scarcity, and the scalability of the algorithm on large-scale datasets require in-depth investigation.

Despite the significant achievements of this study, there are still some limitations that need attention. First, the diversity and complexity of the datasets are insufficient, which may limit the model’s adaptability in real-world logistics distribution. Second, the model has relatively high computational resource demands, particularly for the large-scale training involved in meta-reinforcement learning, which could affect the scalability and practical application of the method. Additionally, this research primarily focuses on logistics path optimization and resource management, and its applicability in other related fields, such as inventory management and delivery time window optimization, has not been fully validated. Finally, there is room for improvement in the model’s real-time adaptability and computational efficiency in dynamic environments, and its interpretability is relatively weak, especially with regard to the specific role of the attention mechanism in the Transformer model’s decision-making process, which still needs further clarification.

To address these limitations, future research will focus on expanding the research dimensions by extending the current two-dimensional path optimization methods to three-dimensional space, particularly for emerging logistics models such as drone delivery. In three-dimensional path optimization, it will be essential to consider multi-dimensional factors such as flight altitude, vertical space constraints, terrain complexity, and drone energy consumption, which will place higher demands on the algorithm’s spatial modeling capabilities. At the same time, we will continue to expand the dataset’s scale and complexity to validate the model’s applicability and robustness, design lightweight models, or introduce distributed computing architectures to reduce computational resource requirements. Additionally, we aim to incorporate more interdisciplinary theories, such as game theory and multi-agent collaborative algorithms, to enhance global optimization capabilities in complex scenarios while also strengthening research on the model’s interpretability. Particularly in three-dimensional dynamic environments, effectively constructing dynamic graph networks based on three-dimensional coordinates and designing graph convolutional units capable of capturing vertical dimension features will be key areas of our continued exploration. Through ongoing theoretical innovation and algorithm iteration, we aim to provide more advanced and flexible path optimization solutions for the intelligent logistics field, offering strong technical support for practical application scenarios such as urban emergency supply distribution, medical material transport in remote areas, and disaster relief.

In summary, this study proposes a method that integrates Graph Neural Networks, Transformer models, and meta-reinforcement learning for optimizing path planning and resource management in logistics distribution. The research has achieved significant results both theoretically and in practical applications, providing a new solution for the intelligent logistics distribution field. As technology advances and methods improve in future research, this field will present broader application prospects. This study not only provides strong support for technological progress in related areas but also outlines the direction for subsequent research, laying the foundation for further enhancing logistics distribution efficiency and resource optimization levels.

## Figures and Tables

**Table 1:** Comparison of route optimality, delivery rate, and resource utilization indicators based on different methods on SCLP and DSCB datasets. The best results are shown in bold.

Model	SCLP Dataset [46]			DSCB Dataset [47]		
	**Route Optimality (%)**	**Delivery Rate (%)**	**Resource Utilization (%)**	**Route Optimality (%)**	**Delivery Rate (%)**	**Resource Utilization (%)**
Hu, Wu-Chih et al. [50]	85.74	85.10	85.68	83.31	83.27	83.66
Al Theeb, Nader et al. [51]	86.78	86.11	87.26	83.97	84.07	84.66
Islam, Md Anisul et al. [52]	87.50	86.16	87.34	84.37	84.55	84.96
Joe, Waldy et al. [53]	89.13	87.28	89.03	84.86	86.18	86.84
Huang, Shan-Huen et al. [54]	89.22	87.82	89.13	85.19	86.31	86.90
Delarue, Arthur et al. [55]	90.98	89.50	89.55	86.71	88.03	87.01
Ours	91.24	90.25	90.02	87.05	88.22	87.64

**Table 2:** Comparison of route optimality, delivery rate, and resource utilization indicators based on different methods on Solomon and SCG datasets.

Model	Solomon Dataset [48]			SCG Dataset [49]		
	**Route Optimality (%)**	**Delivery Rate (%)**	**Resource Utilization (%)**	**Route Optimality (%)**	**Delivery Rate (%)**	**Resource Utilization (%)**
Hu, Wu-Chih et al. [50]	86.62	86.79	86.71	84.11	84.53	84.12
Al Theeb, Nader et al. [51]	87.07	86.95	87.13	84.57	85.03	84.74
Islam, Md Anisul et al. [52]	89.03	89.30	87.25	86.84	86.71	86.60
Joe, Waldy et al. [53]	89.85	89.76	87.84	87.15	86.90	86.78
Huang, Shan-Huen et al. [54]	90.48	92.25	90.11	88.14	89.33	88.72
Delarue, Arthur et al. [55]	91.78	92.64	90.32	90.07	90.12	90.97
Ours	92.29	93.12	90.71	90.75	90.44	91.58

**Table 3:** Comparison of training time, inference time, and parameter indicators based on different methods on SCLP and DSCB datasets.

Model	SCLP Dataset [46]			DSCB Dataset [47]		
	**Training Time (s)**	**Inference Time (ms)**	**Parameters (M)**	**Training Time (s)**	**Inference Time (ms)**	**Parameters (M)**
Hu, Wu-Chih et al. [50]	52.72	145.22	298.22	56.40	159.57	308.33
Al Theeb, Nader et al. [51]	47.80	139.56	291.72	51.85	155.53	303.32
Islam, Md Anisul et al. [52]	43.49	135.05	279.61	49.22	148.87	294.72
Joe, Waldy et al. [53]	40.52	131.98	270.37	45.85	145.44	280.64
Huang, Shan-Huen et al. [54]	38.51	125.22	263.62	41.24	140.68	274.47
Delarue, Arthur et al. [55]	33.85	119.88	250.66	38.07	137.66	264.07
Ours	30.70	113.54	240.48	34.67	135.19	254.34

**Table 4:** Comparison of training time, inference time, and parameter indicators based on different methods on Solomon and SCG datasets.

Model	Solomon Dataset [48]			SCG Dataset [49]		
	**Training Time (s)**	**Inference Time (ms)**	**Parameters (M)**	**Training Time (s)**	**Inference Time (ms)**	**Parameters (M)**
Hu, Wu-Chih et al. [50]	45.87	141.68	288.44	46.69	149.49	300.15
Al Theeb, Nader et al. [51]	42.93	136.61	278.29	43.90	146.30	289.87
Islam, Md Anisul et al. [52]	40.39	132.05	269.29	41.43	142.42	276.79
Joe, Waldy et al. [53]	35.89	125.21	259.02	37.03	140.22	262.48
Huang, Shan-Huen et al. [54]	33.01	119.44	251.59	32.78	134.14	250.95
Delarue, Arthur et al. [55]	30.01	113.65	243.29	30.75	128.86	243.40
Ours	25.49	110.32	235.52	26.59	122.50	234.87

**Table 5:** Comparison of route optimality, delivery rate, and resource utilization indicators of different modules based on SCLP and DSCB datasets.

Model	SCLP Dataset [46]			DSCB Dataset [47]		
	**Route Optimality (%)**	**Delivery Rate (%)**	**Resource Utilization (%)**	**Route Optimality (%)**	**Delivery Rate (%)**	**Resource Utilization (%)**
baseline	60.11	61.02	60.65	59.63	58.77	60.38
+ gnn	75.37	75.60	77.55	73.28	74.43	74.87
+ meta-rl	85.24	85.53	85.74	82.81	83.14	86.21
+ gnn meta-rl	91.33	92.07	92.16	89.17	91.03	89.58

**Table 6:** Comparison of route optimality, delivery rate, and resource utilization indicators of different modules based on Solomon and SCG datasets.

Model	Solomon Dataset [48]			SCG Dataset [49]		
	**Route Optimality (%)**	**Delivery Rate (%)**	**Resource Utilization (%)**	**Route Optimality (%)**	**Delivery Rate (%)**	**Resource Utilization (%)**
baseline	62.92	61.43	63.33	60.98	61.29	62.25
+ gnn	77.94	77.38	75.10	75.13	76.55	75.32
+ meta-rl	87.46	86.54	88.67	86.96	86.42	87.42
+ gnn meta-rl	92.36	91.62	90.98	92.12	92.98	92.46

**Table 7:** Comparison of training time, inference time and Parameters of different modules based on SCLP and DSCB datasets.

Model	SCLP Dataset [46]			DSCB Dataset [47]		
	**Training Time (s)**	**Inference Time (ms)**	**Parameters (M)**	**Training Time (s)**	**Inference Time (ms)**	**Parameters (M)**
baseline	54.68	144.61	267.18	56.13	156.32	289.43
+ gnn	48.40	134.41	258.99	51.13	144.02	280.04
+ meta-rl	45.72	126.13	238.43	48.19	139.95	256.46
+ gnn meta-rl	42.22	110.85	224.32	44.39	111.03	233.83

**Table 8:** Comparison of training time, inference time, and parameter indicators of different modules based on Solomon and SCG datasets.

Model	Solomon Dataset [48]			SCG Dataset [49]		
	**Training Time (s)**	**Inference Time (ms)**	**Parameters (M)**	**Training Time (s)**	**Inference Time (ms)**	**Parameters (M)**
baseline	50.72	143.94	267.83	50.39	143.34	262.59
+ gnn	47.78	135.03	252.29	47.89	135.50	253.04
+ meta-rl	43.08	124.31	232.81	42.89	125.52	225.71
+ gnn meta-rl	40.50	109.17	218.57	39.97	113.39	222.32

**Table 9:** Comparison of robustness test results of supply chain path optimization models under extreme scenarios.

Test Scenario	Evaluation Metrics	Current Method Performance	Traditional Method Performance	Remark
**50% of the path nodes** **are interrupted**	RUR	75%	50%	GNN updates topology in real time, Meta-RL quickly adjusts
	Route Optimality	68%	45%	Still able to generate a feasible path
**A sudden increase in** ** demand of 300% (peak load)**	OTD	82%	60%	Resource scheduling strategies need to be further optimized
	Average response time (s)	30s	120s	Significant real-time dynamic adjustment capabilities
**Adversarial attack** ** (node demand tampering)**	Path planning quality retention rate	89%	65%	Robust Optimization Based on Attention Entropy Constraint
**Adversarial Attack** ** (Edge Weight Tampering)**	Path planning quality retention rate	85%	58%	Anomaly detection module effectively filters noise
**Security policy** ** backup (CBF enabled)**	System crash rate	2%	15%	Control barrier functions to prevent high-risk decisions
	Recovery time (minutes)	2.5	10	Automatically switch to conservative rules

## Data Availability

All datasets utilized in this article are derived from publicly accessible datasets. The specific origins of each dataset have been meticulously referenced within the text, and source links for each dataset are provided in the submission materials to facilitate convenient access and verification by readers. Should there be any inquiries concerning the datasets or if additional information is required, please do not hesitate to contact the authors of this article.
